# Beyond Epilepsy Control: Repurposing Antiepileptic Drugs in Central Nervous System Tumor Therapy

**DOI:** 10.3390/cells15050409

**Published:** 2026-02-26

**Authors:** Haochen Zhao, Qian Jiang, Quanji Wang, Zihan Wang, Yimin Huang, Ting Lei

**Affiliations:** 1 Sino-German Neuro-Oncology Molecular Laboratory, Tongji Hospital, Tongji Medical College, Huazhong University of Science and Technology, Wuhan 430030, China; u201810325@hust.edu.cn (H.Z.); d202382335@hust.edu.cn (Q.J.); quanjiwang@tjh.tjmu.edu.cn (Q.W.); zihanwang@hust.edu.cn (Z.W.); yimin.huang@tjh.tjmu.edu.cn (Y.H.); 2 Department of Neurosurgery, Tongji Hospital, Tongji Medical College, Huazhong University of Science and Technology, Wuhan 430030, China; 3 Hubei Key Laboratory of Neural Injury and Functional Reconstruction, Huazhong University of Science and Technology, Wuhan 430030, China

**Keywords:** antiepileptic drugs, drug repurposing, central nervous system tumors, metabolic reprogramming, epigenetic regulation, endoplasmic reticulum stress, ion homeostasis, tumor immune microenvironment

## Abstract

Antiepileptic drugs (AEDs) are primarily indicated for controlling epileptic seizures. However, accumulating clinical evidence suggests that their benefits in patients with central nervous system (CNS) tumors extend beyond seizure management. Emerging evidence indicates that AEDs possess direct antitumor activity independent of their antiepileptic effects, highlighting a promising novel direction for CNS tumor therapy. This review elucidates the multifaceted antitumor mechanisms of classic (e.g., valproic acid and levetiracetam) and novel (e.g., cannabidiol) AEDs, including their impacts on metabolic reprogramming, epigenetic regulation, endoplasmic reticulum stress and unfolded protein response (ERS-UPR), ion homeostasis, and the tumor immune microenvironment (TIME) to provide new insights and a theoretical basis for developing multitarget therapeutic strategies.

## 1. Introduction

Epilepsy represents a common complication of CNS tumors, with incidence rates varying significantly depending on the tumor type [1]. For instance, 30–90% of glioma patients experience epileptic seizures [2], approximately two-thirds of whom develop epilepsy at diagnosis and the remaining third during treatment [3]. The incidence of epilepsy in meningioma patients is approximately 26% [4]. While among patients with brain metastases, 14–17% develop epilepsy [5], with melanoma metastases being the most prominent [6]. From an anatomical perspective, tumors involving the temporal and frontal lobes exhibit a higher propensity to induce epilepsy due to their proximity to the limbic system and cortical excitatory circuits [1,7]. Tumor tissues trigger seizures through compression and inflammation of normal brain tissue, as well as various molecular mechanisms (e.g., glutamate-mediated excitotoxicity [8], abnormal synaptic connections [9,10], ion homeostasis imbalance [11,12], and specific gene mutations [13,14]). Seizures during the CNS tumor course profoundly affect patients’ consciousness, motor function, treatment, prognosis, quality of life, and survival rate [15,16,17]. Therefore, the management of tumor-related epilepsy is as important as antitumor treatment and is crucial for improving patients’ overall condition.

AEDs have traditionally been used for seizure management, with research predominantly focused on their antiepileptic mechanisms. However, accumulating evidence has demonstrated that patients with CNS tumors may derive benefits from AEDs that extend beyond seizure alleviation. With advancing research, additional mechanisms of AEDs, independent of their antiepileptic effects, have been uncovered. Beyond regulating electrical excitability, AEDs’ effects at the cellular and molecular levels may overlap with those of certain chemotherapeutic agents [18]. This evidence positions AEDs as promising candidates for repurposing—from the prevention and treatment of complications to combination therapies or even standalone antitumor strategies for CNS tumors.

This review discusses the antitumor mechanisms of classic (e.g., valproic acid and levetiracetam) and novel (e.g., cannabidiol) AEDs and their repurposing potential, covering five core mechanistic dimensions: 1. Metabolic reprogramming; 2. Epigenetic regulation; 3. ERS-UPR; 4. Ion homeostasis; and 5. TIME.

## 2. Mechanisms of AEDs Against CNS Tumors

**Figure 1:**
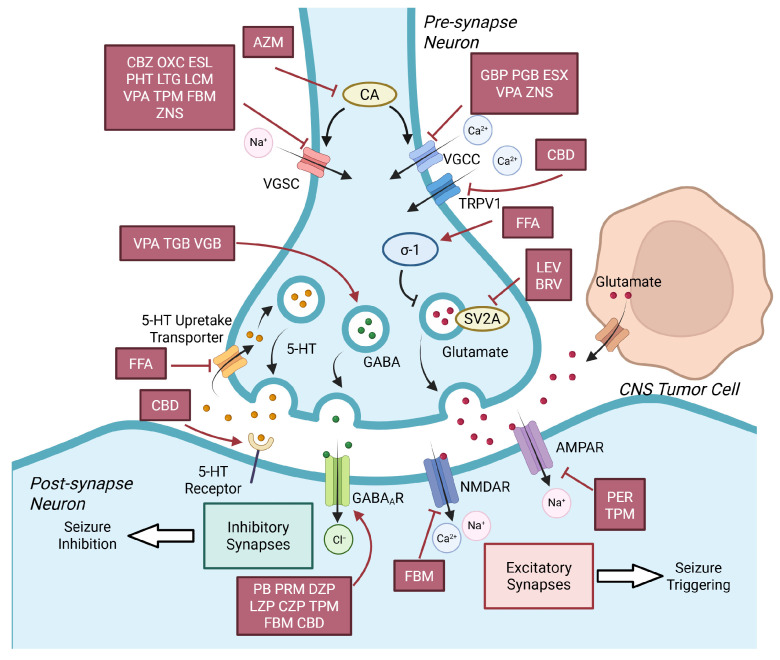
Mechanism demonstration of AEDs’ seizure control targets. VGSC blockers (CBZ, OXC, ESL, PHT, LTG, LCM, VPA, TPM, FBM and ZNS), VGCC blockers (GBP, PGB, ESX, VPA, ZNS), and TRPV1 blocker (CBD) inhibit influx of Na^+^ and Ca^2+^ thus suppress the excitement and conduction of neurons. Glutamate secreted by pre-synapse neurons and CNS tumor cells triggers epileptic seizures by activating AMPAR and NMDAR on post-synapse neurons. LEV, BRV and FFA hinder the glutamate secretion of pre-synapse neurons as LEV and BRV directly bind to SV2A and FFA activates σ-1 receptor. Inhibition of AMPAR (by PER and TPM) and NMDAR (by FBM) lowers down glutamine’s excitatory effect on post-synapse neurons. GABA and 5-HT work for inhibitory synapses and pose antiseizure properties. VPA, TGB and VGB promote secretion of GABA. PB, PRM, DZP, LZP, CZP, TPM, FBM and CBD promote GABA_A_R. FFA elevates concentration of 5-HT in synaptic cleft by blocking 5-HT reuptake transporters, and CBD activates 5-HT receptor, thus magnifies 5-HT’s inhibition on neuron excitability. Abbreviations: CNS, Central nervous system; CBZ, Carbamazepine; OXC, Oxcarbazepine; ESL, Eslicarbazepine; PHT, Phenytoin; LTG, Lamotrigine; LCM, Lacosamide; VPA, Valproic acid; TPM, Topiramate; FBM, Felbamate; ZNS, Zonisamide; VGCC, Voltage-gated calcium channel; CBD, Cannabidiol; TRPV1, Transient receptor potential vanilloid 1; GBP, Gabapentin; PGB, Pregabalin; ESX, Ethosuximide; 5-HT, Serotonin; FFA, Fenfluramine; GABA, γ-aminobutyric acid; GABA_A_R, GABA-A receptor; TGB, Tiagabine; VGB, Vigabatrin; GABA-A receptor; PB, Phenobarbital; PRM, Primidone; DZP, Diazepam; LZP, Lorazepam; CZP, Clonazepam; SV2A, Synaptic vesicle glycoprotein 2A; LEV, Levetiracetam; BRV, Brivaracetam; NMDAR, N-methyl-D-aspartic acid receptor; AMPAR, α-amino-3-hydroxy-5-methyl-4-isoxazolepropionic acid receptor; PER, Perampanel.

The diverse pharmacological mechanisms of AEDs, which form the basis of their efficacy and classification, primarily target neuronal excitability through three core dimensions: ion channel regulation, neurotransmitter system modulation, and novel target intervention. It is precisely this mechanistic diversity that provides the molecular basis for their observed cross-field antitumor effects, as many of these neuronal targets are also aberrantly expressed or functionally implicated in CNS tumors. The primary molecular targets and mechanisms of these AEDs are summarized in Table 1 and visualized in Figure 1.

At the ion channel level, voltage-gated sodium channels (VGSCs) are classic therapeutic targets. Drugs such as Carbamazepine (CBZ) [19] and Oxcarbazepine (OXC) [20] exert antiepileptic effects by regulating these channels, while Lacosamide (LCM) [21] acts via a unique mechanism of selectively enhancing the slow inactivation of sodium channels. Regulating voltage-gated calcium channels (VGCCs) is equally crucial: Ethosuximide (ESX) treats absence seizures by blocking T-type calcium channels in thalamic neurons [22,23], whereas Gabapentin (GBP) [24,25] and Pregabalin (PGB) [26] reduce neurotransmitter release by binding to the α2δ-1 auxiliary subunit of VGCCs.

At the neurotransmitter system level, core strategies include enhancing γ-aminobutyric acid (GABA)-ergic inhibition and attenuating glutamatergic excitation: Phenobarbital (PB) [27], Clonazepam (CZP) [28], and Stiripentol (STP) [29] act positively on GABAA receptors, thereby enhancing inhibitory neurotransmission; Valproic Acid (VPA) enhances seizure inhibition through multiple pathways, such as promoting GABA synthesis and inhibiting its degradation [30]; Perampanel (PER), as an AMPA receptor (AMPAR) antagonist, directly blocks excitatory synaptic transmission to suppress seizures [31].

Levetiracetam (LEV) [32] and Brivaracetam (BRV) [33], which target synaptic vesicle glycoprotein 2A(SV2A), exemplify a novel mechanism of action. Topiramate (TPM) [34] and Felbamate (FBM) [35] exert broad-spectrum antiepileptic activity through synergistic multitarget effects (regulating sodium channels, and the GABAergic and glutamatergic systems). Fenfluramine (FFA), a newly approved AED, provides a novel therapeutic option for refractory epilepsies such as Dravet syndrome by modulating the serotonergic (5-HT) system and σ-1 receptors [36,37]. Acetazolamide (AZM), as a classic carbonic anhydrase (CA) inhibitor, confers antiepileptic effects by blocking the rapid conversion equilibrium between CO_2_, HCO_3_^−^, and H^+^ to regulate intracellular pH, thereby indirectly inhibiting VGSCs and VGCCs to reduce the excitability [38].

Cannabidiol (CBD), a relatively newly utilized AED without psychoactivity, exerts antiseizure effects through multitarget synergy: it desensitizes transient receptor potential vanilloid 1 (TRPV1) channels to reduce calcium influx, enhances GABAergic inhibition, and acts as an inverse agonist of 5-HT receptors to stabilize neuronal excitability [39]. From traditional ion channel regulation to modern synaptic protein and receptor targeting, the continuous expansion of AED mechanisms has greatly promoted precision medicine in epilepsy treatment. While early research predominantly focused on enhancing antiepileptic efficacy, recent breakthroughs have uncovered the direct antitumor activity of AEDs independent of their epilepsy-modulating effects.

**Table 1:** Targets of AEDs for antiepilepsy treatment.

Main Mechanism	AED	Target	References
Sodium Channel Blocker	Carbamazepine (CBZ)	VGSC	[19]
	Oxcarbazepine (OXC)	VGSC	[20]
	Eslicarbazepine (ESL)	VGSC	[40]
	Phenytoin (PHT)	VGSC	[41]
	Lamotrigine (LTG)	VGSC	[42]
	Lacosamide (LCM)	VGSC	[43]
Calcium Channel Blocker	Gabapentin (GBP)	VGCC	[44]
	Pregabalin (PGB)	VGCC	[44]
	Ethosuximide (ESX)	VGCC (T-type)	[22]
SV2A Ligand	Levetiracetam (LEV)	SV2A	[32]
	Brivaracetam (BRV)	SV2A	[33]
GABAergic Agonist	Phenobarbital (PB)	GABA-A receptor	[27]
	Primidone (PRM)	GABA-A receptor	[45]
	Diazepam (DZP)	GABA-A receptor	[46]
	Lorazepam (LZP)	GABA-A receptor	[28]
	Clonazepam (CZP)	GABA-A receptor	[28]
	Tiagabine (TGB)	GABA Transporter 1 (GAT-1)	[47]
	Vigabatrin (VGB)	GABA transaminase	[48]
AMPA Antagonist	Perampanel (PER)	AMPA	[31]
Multitarget or Novel targets	Valproic Acid (VPA)	VGSC, VGCC (T-type), GABAergic system, HDAC	[30,49]
	Topiramate (TPM)	VGSC, AMPA, GABA-A receptor, CA	[50]
	Felbamate (FBM)	VGSC, NMDA, GABA-A receptor	[35]
	Zonisamide (ZNS)	VGSC, VGCC (T-type)	[51]
	Fenfluramine (FFA)	Inhibition of 5-HT re-uptake, activation of σ-1 receptor	[37,52]
	Acetazolamide (AZM)	CA	[38]
	Cannabidiol (CBD)	TRPV1, GABAergic system, 5-HT receptor	[39]

Abbreviations: AED, Antiepileptic drug; VGSC, Voltage-gated sodium channel; VGCC, Voltage-gated calcium channel; SV2A, Synaptic vesicle glycoprotein 2A; GABA, γ-aminobutyric acid; AMPA, α-amino-3-hydroxy-5-methyl-4-isoxazolepropionic acid receptor; NMDA, N-methyl-D-aspartic acid; HDAC, histone deacetylase; CA, Carbonic anhydrase; 5-HT, Serotonin; TRPV1, Transient Receptor Potential Vanilloid 1.

### 2.1. Metabolic Reprogramming

#### 2.1.1. Glycolysis Inhibition

A defining feature of tumor metabolism is the preferential utilization of anaerobic glycolysis for energy production even in the presence of oxygen, a phenomenon termed the Warburg effect [53]. Although less efficient in ATP yield per glucose molecule than oxidative phosphorylation, the high rate of glycolysis enables rapid ATP accumulation, meeting the energy demands of rapidly proliferating tumor cells [54]. Concurrently, lactate accumulated via the Warburg effect promotes glioblastoma (GBM) growth and progression by supplying energy and remodeling the tumor microenvironment [55,56], and is positively correlated with poor prognosis [57]. GBM achieves these effects by upregulating molecular transporters (GLUT-1 [58], GLUT-3 [59], MCT-1 [60]) and metabolism-related enzymes (HK-2 [61], PKM-2 [62,63] LDHA [64]). Inhibition of these molecules can significantly suppress the Warburg effect and impair GBM survival, highlighting their potential as therapeutic targets [58,65,66,67,68,69].

**Figure 2:**
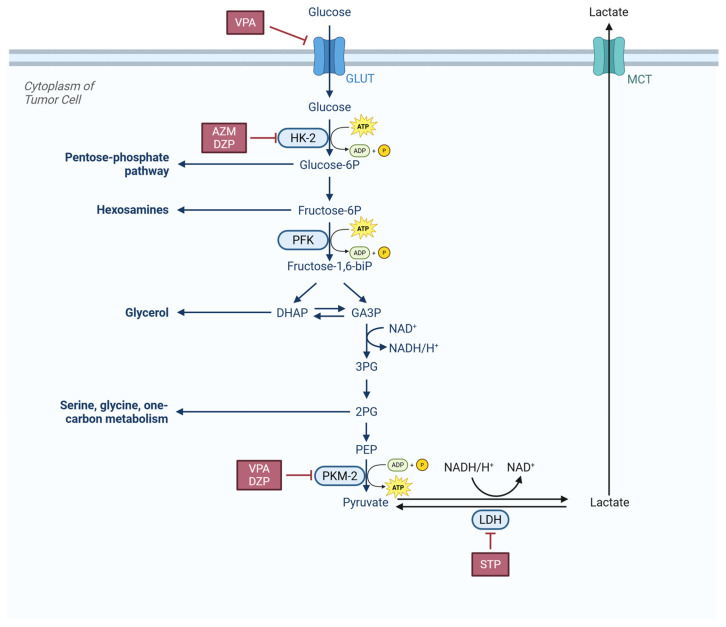
CNS tumor cells upregulate several molecule transporters (GLUT, MCT) and metabolic enzymes (HK, PFK, PKM, LDH), featuring aerobic glycolysis and lactate secretion (Warburg effect). VPA, AZM, DZP and STP inhibit some of these transporters and enzymes to disrupt tumor cells’ glycolysis metabolism. Abbreviations: VPA, Valproic acid; AZM, Acetazolamide; DZP, Diazepam; STP, Stiripentol; GLUT, Glucose Transporter; HK-2, Hexokinase-2; PFK, Phosphofructokinase; DHAP, Dihydroxyacetone phosphate; GA3P, Glyceraldehyde-3-phosphate; 3PG, 3-phosphoglycerate; 2PG, 2-phosphoglycerate; PEP, Phosphoenolpyruvate; PKM-2, Pyruvate kinase isozyme type M2; NAD, Nicotinamide adenine dinucleotide; MCT, Monocarboxylate transporter.

For example, as shown in Figure 2, VPA can suppress tumor survival by inhibiting GLUT-1, thereby reducing glucose uptake [70]. Furthermore, Li et al. (2021) demonstrated that VPA interfered with tumor cell glycolysis by inhibiting pyruvate kinase isozyme type M2 (PKM-2) and exerted a therapeutic effect [71,72], suggesting its therapeutic potential for gliomas characterized by high PKM-2 expression.

DZP can enhance the antitumor effect of lonidamine. By synergistically inhibiting hexokinase (HK) to block tumor cell glycolysis, it disrupts the balance of tumor energy metabolism, significantly enhancing the cytotoxicity of lonidamine on glioma cells and reinforcing growth inhibition [73]. This suggests the potential of DZP in combination antitumor therapy for CNS tumors.

Acetazolamide (AZM) targets and inhibits HK-2 synthesis, a key enzyme in glucose metabolism, thereby markedly disrupting the Warburg effect [74]. This process directly cuts off the main energy supply pathway of tumor cells while reducing metabolic intermediate support for proliferation and invasion.

Stiripentol (STP), as a lactate dehydrogenase (LDH) inhibitor [75], disrupts the Warburg effect and lactic acid metabolism of GBM cells, affecting energy supply and the tumor microenvironment [76]. In drug-resistant models, STP reversed the resistance of U87 cells to temozolomide (TMZ) and exhibited synergistic effects, providing a novel strategy to overcome glioma resistance [77].

#### 2.1.2. Glutamate Metabolism Inhibition

Glutamate, a neurotransmitter, plays a critical role in the pathogenesis of epileptic seizures and CNS tumor progression [78]. GBM cells release glutamate via cystine/glutamate antiporter solute carrier family 7 member 11 (SLC7A11 or xCT) [79,80]. Glutamate accumulated in the tumor microenvironment promotes the proliferation, invasion, and migration of GBM cells themselves by activating receptors such as N-methyl-D-aspartic acid (NMDA) receptors and AMPA receptors on GBM cells; meanwhile, excess glutamate exerts excitotoxicity on surrounding normal neurons, making space for tumor growth and inhibiting antitumor immune response, thereby accelerating tumor malignant progression [81].

LEV regulates neurotransmitter release by binding to SV2A, thereby reducing glutamate levels and alleviates glutamate excitotoxicity. This may be one of the potential mechanisms underlying the survival benefit observed in glioma patients treated with this drug [82,83].

Several clinical studies have suggested that VPA may confer survival benefits to glioma patients when combined with TMZ by indirectly affecting the glutamatergic pathway; however, the precise mechanisms remain to be fully elucidated and warrant further investigation [84].

#### 2.1.3. Carbonic Anhydrase Inhibition

Carbonic anhydrase (CA) is the enzyme responsible for converting carbon dioxide into bicarbonate and protons, regulating pH and metabolism [85]. Its isoforms CA-II, CA-IX, and CA-XII are overexpressed in brain tumors and play important pathophysiological roles [57,86,87,88]. CA-II is specifically overexpressed in GBM stem cells and mediates resistance to temozolomide (TMZ) by regulating cellular metabolism, enhancing invasiveness, and inhibiting autophagy [88]; CA-IX maintains extracellular acidification and activates protumor metabolic pathways, thereby enhancing the invasive phenotype of tumor cells [89]; CA-XII is overexpressed in gliomas and directly drives the invasive phenotype of glioma cells by regulating intracellular pH homeostasis and activating the epithelial–mesenchymal transition (EMT) program, serving as an independent risk factor for poor prognosis [90]. Studies found that inhibiting carbonic anhydrase not only effectively suppressed gliomas but also attenuated their resistance to TMZ [86,89].

TPM and ZNS were found to effectively inhibit carbonic anhydrase isoforms such as CA-II in the brain [91,92,93], and induced intracellular acidosis in tumor cells to kill them [94]. LEV was also identified as a moderate carbonic anhydrase inhibitor, capable of inhibiting CA-IX and CA-XII at the nanomolar level, providing a mechanistic explanation for its therapeutic effect in brain tumors [95,96]. AZM, as an inhibitor of multiple carbonic anhydrase isoforms [91], was shown in several studies to exert antitumor effects by inhibiting these isoforms [74,97,98,99], indicating its potential for direct antitumor activity in CNS tumor treatment.

#### 2.1.4. Effect on Other Targets of Metabolism

Cannabidiol (CBD) can exert antitumor effects by targeting multiple metabolism-related targets in brain tumors. It significantly inhibits tumor proliferation mediated by cannabinoid receptor type 1 (CB1R). Fatty acid metabolic profiling and flow cytometry analysis showed that CBD reduced fatty acid levels in ES-2 cells and significantly inhibited the transcription of genes related to fatty acid uptake and synthesis [100]. On the other hand, CBD inhibits the survival and self-renewal of glioma stem cells by increasing intracellular reactive oxygen species (ROS) levels, and induces the occurrence of mitophagy [101,102]. Studies found that some cells developed drug resistance by upregulating the antioxidant system (e.g., SLC7A11/xCT), while combination of CBD and xCT inhibitors produced a potent synergistic antitumor effect [103]. CBD was found to inhibit NRF2 expression to amplify oxidative stress and induce excessive autophagy through autophagosome accumulation and blocked autophagosome formation, ultimately leading to breast cancer cell death [104]. As NRF2 has also been shown to serve as a therapeutic target for mediating ferroptosis in GBM and attenuating TMZ resistance [105], CBD may treat CNS tumors through the mentioned mechanism.

VPA promotes neuroblastoma differentiation and inhibits proliferation through an SR-mediated, D-serine-independent pathway [106]. Additionally, VPA interferes with fatty acid metabolism by inhibiting fatty acid synthase (FASN), inducing apoptosis in IDH1-mutant glioma cells [107].

#### 2.1.5. Sectional Discussion

A core mechanistic consensus emerging from this section underscores that AEDs exert antitumor effects primarily through three dimensions: glycolysis inhibition, glutamate metabolism inhibition, and CA inhibition. Notably, clinical validation with 54 GBM samples confirms LDHA as potential biomarkers [76], reinforcing these pathways’ translational relevance.

Targeting metabolic reprogramming with AEDs offers a promising therapeutic avenue for CNS tumors. These strategies can bypass conventional chemoresistance (e.g., O (6)-methylguanine-DNA methyltransferase [MGMT] overexpression [108]) and exploit metabolic vulnerabilities (e.g., branched-chain amino acid transport-mTOR crosstalk [109,110]). Additionally, crosstalk between metabolic regulation and the tumor immune microenvironment provides novel insights for combination therapies.

However, existing studies have significant limitations. First, tumor type specificity is insufficient: Li et al. (2021) focused on breast cancer [72], while Supuran (2020) covered broad-spectrum solid tumors (CA IX/XII inhibition) [87], neither addressing CNS-specific features like blood–brain barrier permeability to AEDs [77] or glioma–glial cell metabolic crosstalk [76]. Second, AED class comparisons are lacking: Most literature elaborates on individual agents (e.g., VPA [72] and STP [77]) without clarifying metabolic target differences between classical and novel AEDs. For instance, STP reverses TMZ resistance by inhibiting LDHA [77], but its mechanism has not been compared to LEV’s potential CA IX/XII regulation [87] or CBD-induced ferroptosis. Third, clinical translation evidence is weak: most research remains in vitro/in vivo models, with only Khan et al. (2024) incorporating clinical samples [76]. No studies address how AED clinical dose limitations (e.g., VPA-induced hepatotoxicity [84]) compromise metabolic inhibitory efficacy.

To address these challenges, future studies should focus on integrative analyses of AED effects on tumor metabolism to reveal molecular targets underlying metabolic heterogeneity and advance AEDs from adjuvant to core therapies. Three priority gaps require filling: 1. Supplement LEV/TPM’s regulatory data on CNS tumor to clarify AED subclass-specific metabolic preferences; 2. Evaluate AED combinations with TMZ or immune checkpoint inhibitors to validate synergistic effects (e.g., STP enhancing TMZ sensitivity); 3. Analyze clinical pharmacodynamic–pharmacokinetic correlations between AED plasma concentrations and metabolic biomarkers to optimize dosing regimens.

Collectively, these advancements will refine our understanding of AEDs as repurposed metabolic modulators and strengthen their translational potential for CNS tumor treatment.

### 2.2. Epigenetic Regulation

Epigenetics investigates reversible changes in gene expression (without DNA sequence alterations), primarily encompassing DNA methylation, histone modification, chromatin remodeling, and non-coding RNA regulation [111]. These modifications play crucial roles in tumorigenesis, progression, and therapy resistance. Preclinical studies have confirmed that distinct histone modifications and epigenetic alterations modulate cell viability, inhibit tumor growth, differentiation, and apoptosis, which are crucial for the invasion and metastasis of tumor cells.

#### 2.2.1. DNA Methylation Inhibition

**Figure 3:**
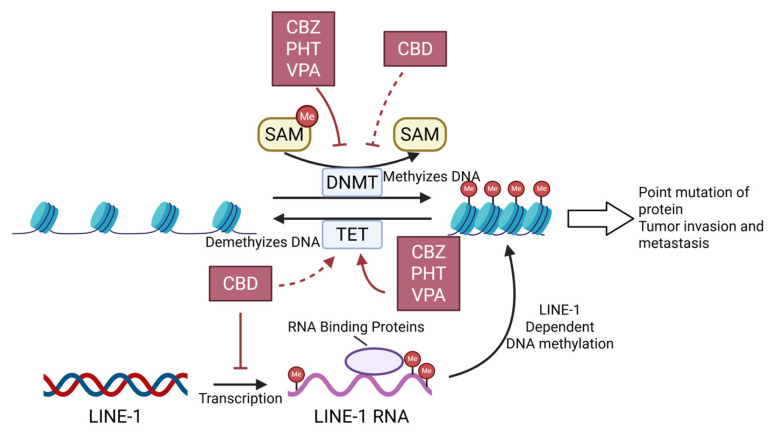
CBZ, PHT and VPA hinder methylation of DNA bidirectionally by inhibiting DNMT and activating TET. CBD hinders L-1 dependent DNA methylation by inhibiting LINE-1′s transcription. Molecular docking indicates CBD’s affinity to DNMT1 and TET1, requiring experimental validation. Abbreviations: CBZ, Carbamazepine; PHT, Phenytoin; VPA, Valproic acid; CBD, Cannabidiol; Me, Methyl group; SAM, S-adenosylmethionine; DNMT, DNA methyltransferase; TET, Ten-eleven translocation methylcytosine dioxygenase; LINE-1, Long interspersed element-1.

DNA methylation (DNAm) is a crucial mechanism regulating chromatin remodeling and gene expression [112]. It is characterized by the transfer of a methyl group from S-adenosylmethionine (SAM) to the C5 position of cytosine, forming 5-methylcytosine (5mC) [113] as shown in Figure 3. In CNS tumors, DNAm and mutations in certain histone loci are associated with tumor invasion and metastasis [114].

CBZ, PHT, and VPA were found to bidirectionally regulate DNA methyltransferases (e.g., DNMT1 and DNMT3A) and demethylases (e.g., TET1, TET2, and TET3), significantly reducing global 5-mC levels and remodeling tumor cells methylation [112].

Evidence supporting the effect of CBD on DNAm remains limited. Molecular docking analyses have shown that CBD exhibits high affinity for DNMT1 and TET1 [115,116,117], which may affect the DNA demethylation process. Research conducted by Li et al. (2024) demonstrated that CBD downregulated the transcription of LINE-1 and reduced DNAm, thereby exerting antitumor efficacy [118]. These findings suggest that CBD has the potential to treat CNS tumors by modulating DNAm.

#### 2.2.2. Histone Deacetylase Inhibition

Overexpression of histone deacetylases (HDACs) is frequently reported in various cancers, where they participate in oncogene activation and the tumor suppressor gene silencing. HDAC inhibitors (HDACi) exert effects including attenuating HDAC activity, increasing chromatin acetylation levels, and restoring the function of tumor suppressor genes [119]. In addition, their ability to sensitize tumor cells to chemotherapeutic agents and radiotherapy has sparked interest in their use as adjuvants to enhance the efficacy of currently employed cancer therapies [120].

**Figure 4:**
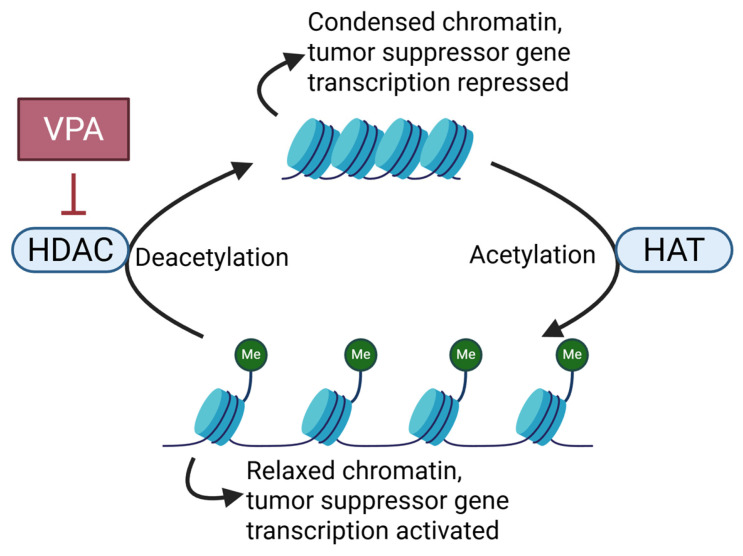
CNS tumor cells suppress expression of tumor suppressor genes through deacetylation of histone. VPA prevents acetylation of histones by inhibiting HDAC and restores expression of tumor suppressor genes. Abbreviations: VPA, Valproic acid; HDAC, Histone deacetylase; HAT, Histone acetyltransferase; Me; Methyl group.

VPA, a well-characterized HDACi (Figure 4), holds promise as a therapeutic agent for CNS tumor treatment [121]. By inducing histone acetylation, VPA promotes the overexpression of cell cycle regulatory genes, such as cyclin-dependent kinase inhibitors (CDKIs) (e.g., p21), leading to tumor cell cycle arrest at various checkpoints and inhibiting cancer cell proliferation [122]. In terms of the epigenetic regulation of tumor suppressor genes, VPA reactivates silenced tumor suppressor genes (SOCS-1/SOCS-3), inhibits cancer cell proliferation, and induces their apoptosis [121].

#### 2.2.3. Regulation on Non-Coding RNAs

Recent advances in understanding non-coding RNA (ncRNA) functions have provided researchers with novel therapeutic targets to repurpose traditional drugs to fight GBM [123]. ncRNAs sustain tumor progression by regulating DNA damage repair, apoptosis, autophagy, cell cycle, metabolic reprogramming, and the Wnt/β-catenin pathway [124], and modulate tumor cells sensitivity to chemoradiotherapy [124,125].

**Figure 5:**
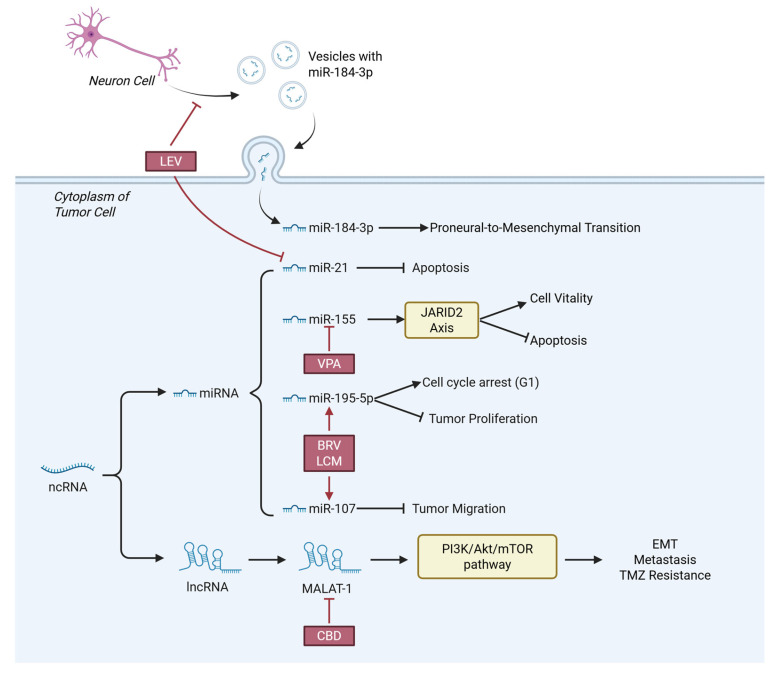
Through the regulation of ncRNA, CNS tumors manage to proliferate, invade and avoid cell death. AEDs can interfere ncRNA to inhibit tumor progression and activate death of tumor cells. Abbreviations: LEV, Levetiracetam; VPA, Valproic acid; BRV, Brivaracetam; LCM, Lacosamide; CBD, Cannabidiol; ncRNA, non-coding RNA; miRNA, microRNA; lncRNA, long non-coding RNA; EMT, Epithelial–mesenchymal transition; TMZ, Temozolomide.

Several AEDs were found to disturb the progression and survival of CNS tumor cells by regulating ncRNAs (Figure 5), providing extra perspectives of CNS tumor treatment.

VPA enhances cancer cell radiosensitivity by downregulating tumor-supportive miRNAs [125]. In glioma cells, the miR-155/JARID2 axis promotes cell viability and suppresses apoptosis [126], while VPA modulates the methylation status of the miR-155 promoter, thereby impairing the functional activity of this oncogenic axis [127].

CBD was found to downregulate MALAT1 to inhibit EMT and metastatic potential in tumor cells [128]. MALAT1 encodes a long non-coding RNA (lncRNA) closely associated with EMT progression and chemoresistance, which activates the PI3K/Akt/mTOR signaling pathway to regulate cancer cell survival, proliferation, and this transformative process [129]. Silencing MALAT1 notably enhances the sensitivity of GBM to temozolomide TMZ chemotherapy [130].

LEV reduces the production of miR-184-3p-enriched exosomes by neurons, thereby inhibiting the proneural-to-mesenchymal transition in glioma stem cells and enhancing cancer radiosensitivity [131]. LEV was also found to downregulate the antiapoptotic factor miRNA-21, disrupting cancer cell apoptotic resistance and ultimately inducing cancer cell death [132]. Another observational study, aiming to identify class-III evidence, showed that the application of LEV throughout standard chemotherapy was associated with a longer median overall survival in individuals with IDH-wildtype glioblastoma [133].

BRV and LCM were found to inhibit the proliferation and migration of glioma cells in vitro by upregulating the expression of miR-195-5p and miR-107, thereby exerting antitumor effects [134], where miR-195-5p of suppressed glioma cell proliferation and induced cell cycle G1 phase arrest [135,136], while miR-107 impaired cell migration ability [137,138].

#### 2.2.4. Sectional Discussion

Current studies indicate that classic AEDs and novel agent CBD exert antitumor effects via DNAm regulation, but evidence exhibits marked “dimensional imbalance”. VPA has the most defined mechanisms: it induces global DNA hypomethylation by inhibiting DNMT1/3A and upregulating TET, and enhances miR-155 promoter methylation to block its suppression of tumor suppressor JARID2, forming a “methylation-miRNA-target gene” axis in glioma [127]. This mechanism is validated across breast cancer and hepatocellular carcinoma [122], with a complete evidence chain and strong cross-cancer applicability. In contrast, CBZ and PHT studies are limited to embryonic HEK293 cells, where they disrupt DNMT/TET balance [112], but lack tumor-specific data, limiting relevance to “AEDs repurposing for CNS tumors”. CBD shows “multitarget but low-consistency” epigenetic effects: in vitro/in silico studies demonstrate direct binding to TET1 or indirect DNMT regulation via CB1 receptors [117]; in NSCLC, it synergizes with CIK cells to reduce LINE-1 methylation [118]; yet in neural cells, it reverses stress-induced abnormal methylation [117]. CBD research is predominantly cell-based, lacking clinical validation and CNS tumor-specific methylation data, with weaker evidence than VPA.

Existing research has notable limitations and clinical translation gaps: VPA’s methylation regulatory effects have been validated in glioma and breast cancer [122,127], but CBZ and PHT studies are confined to HEK293 embryonic cells, which fail to recapitulate the epigenetic characteristics of CNS tumors (e.g., IDH mutation-related methylation reprogramming in glioblastoma) [112]; CBD research primarily focuses on non-small cell lung cancer and pancreatic cancer [118,128], with its epigenetic effects on brain tumors remaining unclarified. Mechanistically, studies predominantly adopt a “single drug–single methylase” linear regulatory framework, neglecting potential synergistic or antagonistic effects between AEDs (e.g., VPA combined with CBZ) and failing to link methylation changes to core driver pathways of CNS tumors (such as PI3K/Akt and MAPK) [128], resulting in an incomplete mechanistic chain between methylation alterations and tumor suppression. Clinically, all epigenetic studies are based on basic experiments, lacking methylome analysis of tumor tissues from AED-treated patients and failing to establish correlations between key methylation biomarkers (e.g., LINE-1, miR-155) and treatment response [118,127]. Additionally, CBD’s methylation regulatory activity is weaker than that of VPA [117], and the absence of direct comparative data with classic AEDs leads to ambiguous priority ranking of epigenetic effects among different AEDs.

Future research should focus on three aspects: 1. Use CNS tumor-specific models (IDH wildtype/mutant glioblastoma) to explore VPA/CBD-mediated methylation changes at key loci (LINE-1, miR-155) and their association with tumor phenotypes [118,127]. 2. Supplement clinical evidence by analyzing correlations between tumor methylomes and prognosis in AED-treated patients to screen efficacy-monitoring biomarkers. 3. Investigate VPA-CBD synergy via “DNMT inhibition + TET regulation” while avoiding CBZ/PHT’s embryonic toxicity [112].

From a review perspective, de-emphasize CBZ/PHT’s non-tumor model data, highlight VPA’s cross-cancer evidence [122,127] and CBD’s tumor-specific research [118,128], and clarify AED positional differences via “drug–target–tumor type” comparison to align with the review theme.

### 2.3. Endoplasmic Reticulum Stress and Unfolded Protein Response

**Figure 6:**
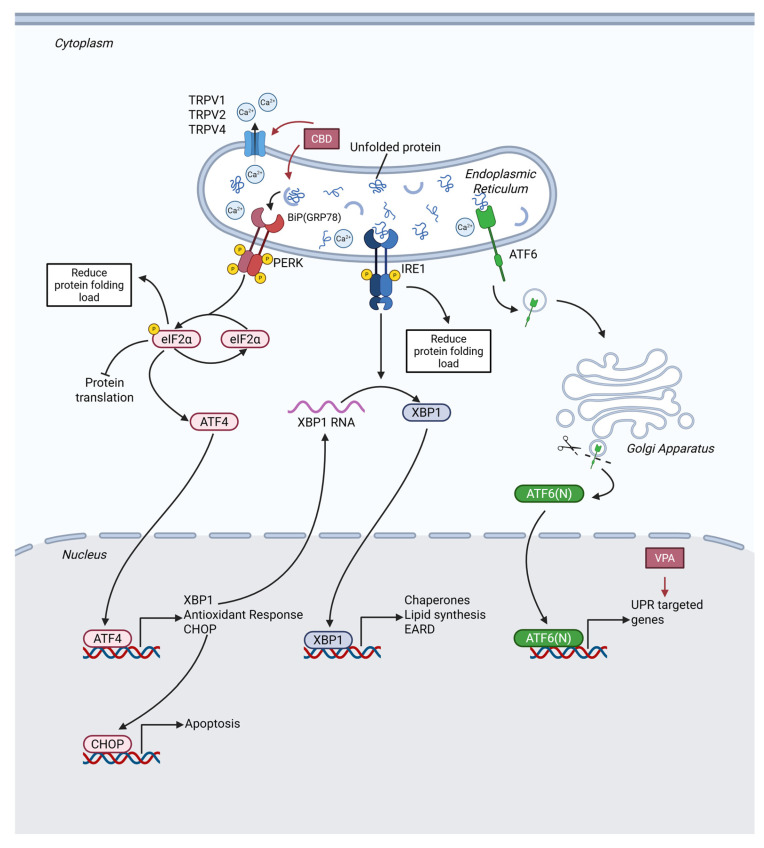
Calcium efflux and unproperly folded protein accumulation are the main motivations of ERS. To regain homeostasis of calcium and protein, UPR pathways (PERK-eIF2α pathway, IRE1-XBP1 pathway and ATF6 pathway) are activated. By promoting excess ERS and disrupting UPR pathways, AEDs divert tumor cells into apoptosis signaling. Abbreviations: CBD, Cannabidiol; VPA, Valproic acid; TRPV, Transient receptor potential vanilloid; BiP, Binding immunoglobulin protein; PERK, Protein kinase-like endoplasmic reticulum kinase; eIF2α, eukaryotic initiation factor 2α; ATF4, Activating transcription factor 4; CHOP, C/EBP homologous protein; IRE1, Inositol requiring enzyme-1; XBP1, X-Box binding protein 1; ERAD, Endoplasmic reticulum-associated degradation; ATF6, Activating transcription factor 6; UPR, Unfolded protein response.

Endoplasmic reticulum stress (ERS) and unfolded protein response (UPR) maintain a dynamic balance in tumor cells, with their functional roles exhibiting a notable double-edged nature. When ERS occurs, as shown in Figure 6, the UPR coordinates protein folding, degradation, and translational regulation by activating the IRE1α, PERK, and ATF6 signaling pathways [139]. In cancer cells, moderate UPR promotes cell survival (e.g., glioblastoma maintains proliferation under hypoxic conditions through GRP78 upregulation [140]); otherwise, sustained or excess ERS triggers apoptosis (e.g., endoplasmic reticulum Ca^2+^ efflux in glioma cells leads to activation of the ATF4/CHOP-dependent death pathway [141]). CNS tumors exhibit more complex regulation of the UPR due to their unique blood–brain barrier and immune microenvironment: the IRE1α/XBP1 pathway is specifically activated in glioma stem cells (GSCs), which is closely associated with TMZ resistance and angiogenesis [142].

#### 2.3.1. CBD’s Effect on ERS and UPR

CBD has exhibited antitumor activity in various tumor models, and its mechanism of action is closely associated with the induction of ERS and UPR [143].

In glioma, CBD acts as an agonist of the TRPV4 ion channel, inducing calcium ion influx thereby activating the ATF4–DDIT3–TRIB3 axis and inhibiting the Akt-mTOR pathway. Additionally, CBD can suppress autophagic flux, leading to the accumulation of the autophagic substrate p62/SQSTM1, while enhancing the expression and activity of E3 ligases (such as Hrd1 and gp78) in the ER-associated degradation (ERAD) machinery [144]. Another study demonstrated that CBD disrupted ER homeostasis and triggered a robust UPR. Specifically, it significantly upregulated key ERS markers such as the chaperone BIP/GRP78, and activates the PERK–eIF2α–ATF4 signaling axis, thereby promoting the expression of the pro-apoptotic transcription factor CHOP [145]. Furthermore, CBD disrupt intracellular calcium homeostasis via the TRPV1/TRPV2 or CB1/CB2 receptors, accompanied by the production of large amounts of ROS. This forms a positive feedback loop with ERS, further amplifying the UPR signal [100,118,146,147]. The dysregulation of protein quality control mechanisms collectively leads to the abnormal accumulation of unfolded or misfolded proteins in the endoplasmic reticulum lumen, thereby triggering ERS and specifically activating IRE1α and PERK, the two major branches of the UPR, ultimately mediating tumor cell apoptosis.

In terms of cell death pathways, CBD regulates the balance of Bcl-2 family proteins via the ATF4–CHOP axis, promotes the expression of the pro-apoptotic protein Bax, activates Caspase-3/9 and PARP cleavage, and executes the mitochondria-dependent apoptotic program [148]. Meanwhile, the UPR induced by CBD can also inhibit the Akt/mTOR pathway through the p8–TRIB3 axis, relieving the inhibition of autophagy, characterized by the conversion of LC3-I to LC3-II and the induction of mitophagy via the PINK1–PRKN pathway [144]. In models such as GBM, CBD further inhibits the cystine/glutamate antiporter SLC7A11 through the combined action of ERS and ROS, leading to impaired glutathione synthesis and GPX4 inactivation, thereby triggering ferroptosis [101]. Additionally, CBD can induce caspase-independent paraptosis, a process dependent on the sustained activation of the ATF4/CHOP pathway and accompanied by downregulation of ALIX protein and cytoplasmic vacuolization [149].

#### 2.3.2. Other AEDs’ Effect on ERS and UPR

The therapeutic potential of VPA against CNS tumors depends on the precise regulation of the ERS-UPR pathway. In a study conducted focusing on GBM, Cattaneo et al. (2014) found that VPA effectively activated the expression of genes associated with the UPR in GBM cell lines and reduced GBM’s viability and aggressiveness by interfering with SEL1L, a regulatory protein in the UPR pathway, further magnified VPA’s antitumor activity [150]. Silencing SEL1L can synergistically enhance the inhibitory effect of VPA on GSC proliferation and self-renewal capacity, induce cells to differentiate into neurons by downregulating the Notch1 signaling pathway, and further amplify ERS-mediated apoptotic effects. This synergistic mechanism provides a new direction for overcoming drug resistance in CNS tumor stem cells. Although direct evidence for VPA regulating ERS in CNS tumors is mainly from a 2014 study, the consistency of its mechanism with subsequent ERS regulation research confirms the core status of this pathway.

As an AED that positively modulates the σ-1 receptor [37], FFA expands the mechanistic landscape of AEDs in regulating ERS in CNS tumors. The σ-1 receptor, localized at the ER-mitochondria contact site, alleviates adaptive ERS responses by regulating calcium homeostasis when activated, while its antagonists enhance CNS tumor cell sensitivity to ERS inducers by abrogating this protective mechanism [151]. Leveraging its antiepileptic pharmacological properties and σ-1 receptor modulatory activity [52], FFA is speculated to regulate the ERS-UPR balance through σ-1 receptor activation: on one hand, it inhibits apoptosis resistance induced by excessive ERS, and on the other hand, it enhances tumor cell sensitivity to therapeutic interventions. This mechanism provides theoretical support for σ-1 receptor-targeted combined with ERS-modulating therapy for CNS tumors.

#### 2.3.3. Sectional Discussion

While the aforementioned studies collectively support the potential of AEDs to target CNS tumors through ERS and UPR signaling, several limitations and inconsistencies in the current evidence base warrant critical consideration.

First, while CBD has emerged as a more extensively studied AED in this context, with multiple recent reports supporting its ERS-mediated antitumor activity [100,101], most evidence relies on in vitro models or non-CNS tumors. For instance, CBD’s ERS effect was demonstrated in colorectal cancer [147] and ovarian cancer [100]. Translating these findings to CNS tumors remains challenging due to the unique blood–brain barrier microenvironment and distinct metabolic features of glioblastoma and GSCs. Furthermore, CBD’s role as a TRPV2 activator [100] introduces complexity in disentangling ERS-specific effects from other parallel signaling cascades [101]. Second, the mechanistic evidence for VPA is notably outdated and lacks recent validation. The core study linking VPA to ERS-UPR in GSCs was published in 2014 [150], with no subsequent studies over the past decade to confirm or extend these findings. This gap raises questions about the reproducibility of VPA’s ERS-modulating effects in contemporary CNS tumor models, especially given advancements in understanding tumor heterogeneity and drug resistance mechanisms. Additionally, VPA’s non-specificity as a histone deacetylase inhibitor complicates the attribution of its cytotoxicity solely to ERS-UPR regulation, as it may exert off-target effects on other pathways that independently influence tumor progression [150]. Third, the proposed mechanism linking FFA to ERS-UPR regulation in CNS tumors remains a theoretical integration rather than empirical fact. FFA’s well-documented activity as a positive modulator of σ-1 receptors [14,37] and provides a plausible molecular link to ERS balance. However, direct evidence demonstrating FFA-induced ERS-UPR modulation in CNS tumor cells is absent. The existing data only confirm FFA’s σ-1 receptor binding in mouse models [37], with no studies investigating its impact on ERS markers (e.g., GRP78, CHOP) or UPR branches (PERK, IRE1α) in glioblastoma or other CNS tumors.

At the clinical level, the ERS and UPR-inducing mechanisms of AEDs represent a unique therapeutic approach for the treatment of CNS tumors, particularly GBM: apoptosis induced by this pathway is independent of p53 status, providing a novel approach to overcoming resistance to traditional therapies in GBM (which has a high incidence of p53 mutations) [152]; glioma cells have an inherent high protein synthesis load, leading to a high basal level of ERS, and are more sensitive to ERS exacerbated by AEDs (such as VPA, CBZ, and ZNS), forming a potential therapeutic window [141]; furthermore, complex crosstalk between ERS and cellular processes such as autophagy opens up new avenues for developing sequential combination therapy strategies. Future studies need to clarify the molecular balance point at which AEDs regulate the switch of UPR from pro-survival to pro-apoptotic, explore the potential of molecules such as GRP78 and CHOP as biomarkers for treatment response, and achieve personalized medication.

### 2.4. Ion Homeostasis

Cellular ion homeostasis is the foundation for maintaining normal life activities, involving processes like proliferation and programmed cell death [153]. Tumor cells reprogram ion homeostasis to support rapid proliferation, apoptosis resistance, and adaption to the harsh microenvironment. Consequently, the disruption of ion homeostasis has emerged as a highly promising novel anticancer strategy [154]. The core lies in specifically disrupting the ion balance within tumor cells, thereby activating various death signaling pathways (e.g., apoptosis, ferroptosis, and autophagy), and regulating antitumor immune responses.

#### 2.4.1. CBD’s Effect on Ion Homeostasis

CBD can induce Ca^2+^ permeation through TRPV2, thereby altering membrane potential and reducing the chemoresistance of glioma cells [155]. Additionally, CBD increases the permeability of the VDAC1 channel, promoting the rapid influx of calcium ions into mitochondria and disrupting calcium homeostasis, further leading to mitochondrial dysfunction characterized by mitochondrial membrane potential perturbation, ROS release, and ATP depletion. Such dysfunction also induces the formation of mitochondrial permeability transition pores (mPTP), mitochondrial swelling, and subsequent cell death [146,156]. Furthermore, CBD downregulates the antiapoptotic protein Bcl2 and the mitochondrial fusion protein Mitofusin-2, while upregulating apoptosis-related proteins (cleaved Caspase-3/8/9). Notably, the use of the ROS scavenger N-acetylcysteine can reverse CBD-induced mitochondrial dysfunction and cell apoptosis [100]. CBD drives the phosphorylation of Bcl-2 by activating the JNK1/2 pathway, thereby disrupting the competitive interaction between BECN1 and Bcl-2 [157], leading to autophagy-mediated cell death, which points out a possibility for the treatment of GBM.

#### 2.4.2. Sectional Discussion

Notably, altering ion homeostasis is the signature mechanism of many classic AEDs. However, research on the anticancer efficacy of AEDs through this signature mechanism has been scarce in recent years (2020–2025). Most contemporary studies on AEDs’ antitumor effects focus on epigenetic regulation or metabolic remodeling, with limited in-depth exploration of ion homeostasis disruption as a core antitumor pathway. Even for AEDs known to modulate ion channels, recent investigations primarily emphasize their antiepileptic efficacy in tumor-related epilepsy rather than direct antitumor effects via ion balance modulation. This research gap hinders the comprehensive understanding of AEDs’ antitumor potential and the development of targeted combination therapies [158].

Critical limitations exist in current research on CBD’s ion homeostasis-mediated antitumor effects. First, the concentration of CBD used in most in vitro studies is significantly higher than the clinically achievable plasma concentration (usually 1–10 μM), raising questions about the translational potential of these findings [100]. This study showed CBD exerted antitumor effects in ovarian cancer cells at 20–60 μM (IC_50_ = 32 μM), which is far above the clinical plasma concentration range. Second, existing studies lack in vivo validation using patient-derived xenograft models, which are more representative of the human tumor microenvironment, making it difficult to confirm whether CBD can effectively disrupt ion homeostasis in intact tumor tissues [156]. Third, the interaction between CBD and other ion channels in tumor cells remains unclear, and whether there is crosstalk with other antitumor pathways (such as epigenetic regulation) requires further investigation [157].

In summary, although CBD’s regulation of ion homeostasis provides a potential antitumor mechanism, current research is limited by insufficient clinical relevance and incomplete mechanism elucidation. Coupled with the overall scarcity of recent studies on AEDs’ antitumor effects through ion homeostasis disruption, future research should focus on optimizing in vitro and in vivo models, exploring the synergy between ion channel modulation and other therapeutic strategies, and conducting clinical trials to validate the efficacy and safety of AEDs targeting tumor ion homeostasis.

### 2.5. Tumor Immune Microenvironment

CNS tumors remodel their surrounding tumor immune microenvironment (TIME) through extensive crosstalk with neighboring cells, affecting immune efficacy and tumor progression [159]. The TIME of GBM is characterized as “profound immunosuppression and dysfunctional infiltration”. Among glioma-associated macrophages (GAMs), IDH-mutant low-grade gliomas are dominated by microglia, while IDH-wildtype high-grade gliomas are mainly infiltrated by macrophages prone to M2 polarization [160,161]; GAMs amplify immunosuppression by secreting anti-inflammatory cytokines, consuming immune nutrients, interacting with glioma stem cells (GSCs), and via the TREM2 pathway [76,162]. T cell infiltration in GBM only accounts for 1–10%, mostly presenting as the terminal exhausted phenotype (Tex) without TCF1^+^ subsets, and NK cell infiltration is less than 2.5% with functional loss [163]. Within GBM TIME, astrocytes recruit GAMs through chemokines and inhibit CD8^+^ T cells [164,165].

**Figure 7:**
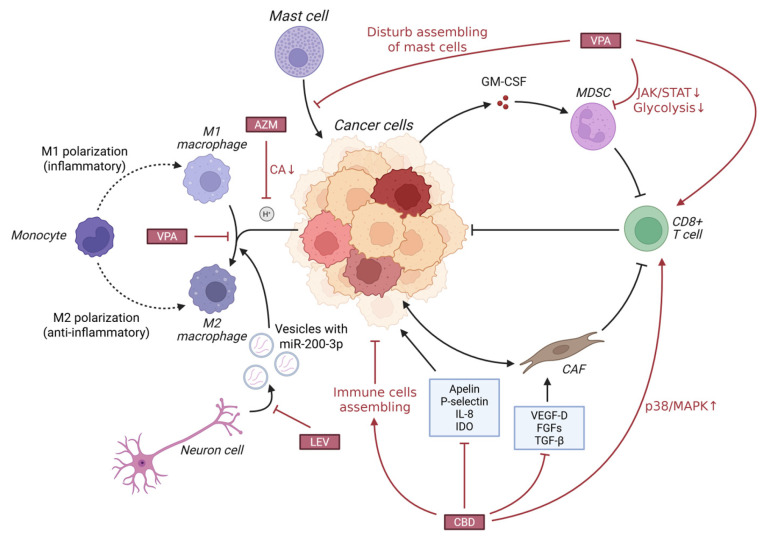
AEDs pose antitumor effects through suppressing protumor microenvironment (M2 macrophage, MDSC, CAF, mast cells) and promoting antitumor microenvironment (M1 macrophage, CD8+ T cells, NK cells). Abbreviations: ↑, increased or activated; ↓, decreased or inhibited; AZM, Acetazolamide; LEV, Levetiracetam; CBD, Cannabidiol; VPA, Valproic acid; CA, Carbonic anhydrase; GM-CSF, Granulocyte-macrophage colony-stimulating factor; MDSC, Myeloid-derived suppressor cell; JAK/STAT, Janus kinase/Signal transducer and activator of transcription; CAF, Cancer-associated fibroblast; IL-8, Interleukin 8; IDO, indoleamine 2,3-dioxygenase; VEGF-D, Vascular endothelial growth factor D; FGF, Fibroblast growth factor; TGF-β, Tumor growth factor β; p38/MAPK, p38 mitogen-activated protein kinase.

Some AEDs have been found to inhibit the immunosuppressive functions of GAMs and Treg cells in the TIME, or enhance the activity of cytotoxic T cells, reverse tumor immunosuppression, and indirectly exert antitumor effects. The mechanisms of several AEDs posing antitumor effects through modulating TIME are visualized in Figure 7.

#### 2.5.1. CBD’s Effect on TIME

CBD exhibits great potential to inhibit tumor progression by remodeling the TIME, transforming the immunosuppressive TIME into an immune-activated phenotype. Studies have shown that its antitumor effect strictly depends on the adaptive immune system: CBD actively recruits CD4+ T cells, CD8+ T cells, B cells, NK cells, and M1-type macrophages to infiltrate the tumor core, and may enhance their functions by activating the p38 mitogen-activated protein kinase (p38/MAPK) signaling pathway in T cells [117,166], which has been verified in orthotopic glioblastoma models. A study conducted by Khodadadi et al. (2023) confirmed that inhaled CBD downregulated pro-angiogenic factors such as Apelin, P-selectin, and IL-8, as well as the immunosuppressive molecule IDO in tumor tissues and enhanced the infiltration of cytotoxic CD8+ T cells, thereby effectively inhibited tumor growth [167]. CBD can also disrupt the microenvironmental network supporting tumor growth by regulating the crosstalk between tumor cells and stromal cells by bidirectionally reprograming the communication between tumor cells and fibroblasts, inhibiting the activation of fibroblasts and the migration/invasion ability of tumor cells, and extensively downregulating the secretion of various pro-angiogenic and pro-proliferative factors such as VEGF-D, FGFs, and TGF-β [168].

#### 2.5.2. VPA’s Effect on TIME

VPA exerts its antitumor efficacy through direct effects on tumor cells as well as by profoundly remodeling the TIME, converting “immune-cold” tumors into “immune-hot” tumors.

VPA downregulate the JAK/STAT signaling pathway and glycolytic metabolic pathway on which myeloid-derived suppressor cells (MDSCs) rely for survival and function, thereby impairing their immunosuppressive capacity and removing obstacles for subsequent immune checkpoint inhibitor therapy [169]. A study by Cai, Z. (2021) [170] found that VPA significantly polarized TAMs from the protumorigenic M2 phenotype to the antitumorigenic M1 phenotype, characterized by the upregulation of M1 markers (e.g., CD86, MHC-II) and the downregulation of M2 markers (e.g., CD163, CD209). Activated M1 macrophages possess potent direct tumor cell phagocytic capacity and can secrete IL-12 to initiate subsequent adaptive immune responses [170]. VPA also remodels innate immunity, creating favorable conditions for T cell activation: IL-12 secreted by M1 macrophages is a key signal for activating CD8^+^ T cells [171]. VPA combination therapy can lead to a significant increase in the number of intratumoral CD8^+^ T cells and the expression of their effector molecule Granzyme B, which can be maintained for a long time after the end of treatment, thereby effectively inhibiting tumor recurrence and forming long-lasting immune memory [170]. VPA can also inhibit the activation and recruitment of mast cells to the tumor site, indirectly impairing their function of promoting tumor progression [172]. In combination immunotherapy, VPA enhances myeloid cell inflammatory signaling pathways such as TREM1 and TLR in responders, thereby amplifying the immune activation effect of anti-PD-L1 agents [169]. And when combined with radiotherapy, VPA induces tumor blood vessels to become sparse and regular—an effect associated with IFN-γ secreted by M1 macrophages helping alleviate tumor hypoxia and promote immune cell infiltration [170].

#### 2.5.3. Other AEDs’ Effect on TIME

AZM, as a CA-IX inhibitor, can reverse the acidic state of the tumor microenvironment. Research demonstrated that when combined with the CHOP regimen, it attenuated hypoxia and acidity in A20 lymphoma tissues; moreover, this combination therapy resulted in a significantly higher number of intratumoral CD3^+^ and CD8^+^ T cell infiltration compared with CHOP monotherapy [173]. This finding confirms that neutralizing the TIME pH through CA inhibition relieves the suppression of T cells and creates a TIME more conducive to immune cell function [174], which could be of use for CNS tumor treatment.

LEV possesses the ability to revert protumorigenic TIME, with a particular focus on counteracting neuron-mediated microglial M2 polarization [175]. Mechanistically, this effect is mediated by LEV’s regulation of neuron-derived exosomal signaling: under hypoxic conditions associated with CNS tumor progression, neurons secrete exosomes enriched in miR-200c-3p, which has been shown to directly promote microglial polarization toward the M2 phenotype via activation of the PTEN/PI3K/Akt pathway [176,177]. By interfering with this exosome-mediated crosstalk between neurons and microglia, LEV disrupts the downstream protumor signaling cascades driven by M2-polarized microglia, thereby mitigating immune tolerance and supporting antitumor immune responses.

#### 2.5.4. Sectional Discussion

While AEDs exhibit promising potential in remodeling the TIME of CNS tumors, critical limitations and context-dependent disparities in their immunomodulatory effects must be acknowledged. First, the antitumor immune activity of CBD, though extensively validated in preclinical models (e.g., HPV-positive HNSCC and orthotopic GBM), is highly dependent on the integrity of the adaptive immune system—its efficacy is completely abrogated in immunodeficient mice (Rag1^−^/^−^ or athymic nude mice), highlighting limited utility in patients with compromised immunity [166,167]. Moreover, CBD’s regulation of TIME is cell type-specific: while it enhances CD4^+^/CD8^+^ T cell and M1 macrophage infiltration in most models, depletion of CD4^+^ T cells unexpectedly exacerbates tumor growth in CBD-treated mice, revealing a fragile balance between pro- and antitumor immune responses that may be disrupted by individual immune status [166]. Second, VPA’s ability to polarize TAMs toward the M1 phenotype and enhance CD8^+^ T cell cytotoxicity is primarily observed in combination with radiotherapy or immunotherapy, with minimal single-agent efficacy in reshaping immunosuppressive TIME [170]. Clinical evidence further indicates that VPA’s immunomodulatory effects are heterogeneous—non-responders to VPA plus avelumab therapy exhibit persistent elevation of IL-8/IL-18, which recruits MDSCs and blunts antitumor immunity, whereas responders show reduced myeloid cell infiltration and enhanced T cell activation [169]. Third, AZM improves T cell infiltration by neutralizing the acidic TIME, but this effect is synergistic only with chemotherapy (e.g., CHOP regimen) and lacks sufficient evidence in CNS tumors specifically, raising questions about its translational relevance given the unique blood–brain barrier and immunosuppressive features of CNS tumor TIME [173]. Additionally, most preclinical studies use high doses of AEDs or syngeneic models with intact immunity, which may not recapitulate the clinical scenario of advanced CNS tumors with exhausted T cells and dense MDSC infiltration.

Collectively, these findings underscore that AED-mediated TIME modulation is not universally effective—its success hinges on tumor type, immune competence, and combination with other therapies—thus emphasizing the need for patient stratification based on immune biomarkers (e.g., IL-8/IL-18 levels, T cell exhaustion status) and further validation in CNS tumor-specific clinical trials. Beyond addressing these limitations, understanding the mechanisms by which AEDs regulate the TIME holds notable clinical significance: it provides a potential strategy for converting “immune-cold” tumors into “immune-hot” tumors by reversing immunosuppressive states to enhance antitumor immunity; TIME modulation profoundly influences the efficacy of radiotherapy, chemotherapy, and immunotherapy; additionally, combining immunotherapy with AEDs may reduce the incidence of immunotherapy-related adverse events and improve treatment safety.

## 3. Overall Discussion

### 3.1. Current Situation and Rationale for Repurposing

In recent years, although significant breakthroughs have been made in tumor therapy, it still faces challenges such as limited efficacy, severe side effects, high development costs, and insufficient clinical conversion rates. Against this backdrop, multi-mechanism research and cross-disease repurposing of clinically approved drugs have garnered significant interest owing to advantages of lower development costs, shorter research periods, and well-established safety profiles.

The clinical management of CNS tumor-related epilepsy provides crucial clinical clues for the antitumor potential of AEDs, prompting researchers to further explore their molecular mechanisms and clinical application possibilities in the treatment of CNS tumors and other system tumors. Significant overlap in the mechanisms between AEDs and antitumor drugs further provides a scientific basis for their repurposing, by regulating core pathways such as tumor metabolic reprogramming, epigenetic modification, endoplasmic reticulum stress, ion homeostasis, and immune microenvironment, AEDs demonstrate great potential as combination therapy agents or monotherapy agents.

Accumulating clinical evidence has further validated this repurposing potential. A recent retrospective observational study [178] explored the association between two AEDs and survival in glioblastoma patients undergoing standard therapy. The findings indicated that LEV use was linked to improved overall survival, while LCM was independently associated with prolonged progression-free survival. Notably, this study provides direct clinical hints for the “non-epilepsy-related” antitumor potential of these AEDs, as the survival benefits persisted after adjusting for seizure status and other confounding factors. However, this retrospective design has inherent limitations, including selection bias and unclear optimal dosage/duration of LEV/LCM, highlighting the need for prospective trials to validate these findings.

Significant differences exist in the strength of antitumor evidence, core mechanisms, and application potential among various AEDs (Table 2). Among classic AEDs, VPA demonstrates the highest translational potential due to its multidimensional synergistic mechanisms and well-established clinical application foundation. LEV, with its well-built safety profile and favorable blood–brain barrier permeability, serves as a preferred adjuvant agent for combination therapy. CBD, a novel AED, exhibits remarkable efficacy in inducing tumor cell death through multiple pathways; however, the gap between clinically achievable concentrations and in vitro effective concentrations limits its direct clinical application. These heterogeneous characteristics not only reflect the progress and limitations of current research but also provide a clear direction for the clinical translation of drug repurposing.

### 3.2. Challenges and Limitations

Repurposing AEDs as adjuvant therapeutic agents for CNS tumors requires overcoming multiple challenges.

First, different AEDs exert heterogeneous antitumor mechanisms, and tumor cell heterogeneity may result in variations in drug sensitivity. Selecting appropriate AEDs based on molecular characteristics requires further clarification. Notably, this heterogeneity is also reflected in the striking disparity in the strength of antitumor evidence among different AEDs—some agents (e.g., VPA, LEV, CBD) have accumulated relatively robust preclinical and preliminary clinical data, while others (e.g., CBZ, PHT, FFA) lack sufficient tumor-specific validation. This discrepancy stems from multiple factors: 1. Mechanistic overlap with established anticancer pathways: VPA’s HDAC inhibition, CBD’s regulation of ERS and TIME, and LEV’s blood–brain barrier permeability align with well-recognized antitumor targets, driving intensive research; in contrast, AEDs primarily acting on ion channels (e.g., CBZ, LCM) have been less explored for their antitumor potential due to historical focus on their antiepileptic effects. 2. Clinical availability and safety profiles: VPA and LEV have long clinical histories and favorable tolerability, facilitating translational research, while novel AEDs like CBD face regulatory and accessibility barriers that hinder large-scale studies. 3. Tumor type-specific relevance: Most research focuses on high-incidence CNS tumors (e.g., glioblastoma), leading to sparse data for rare subtypes (e.g., meningioma, brain metastases), exacerbating evidence imbalance.

Second, the antitumor effects are dose-dependent, while dose-related toxicities (e.g., cognitive impairment, VPA-induced hepatotoxicity) limit clinically applicable doses, making the balance between efficacy and toxicity a key challenge to clinical translation. This issue is further compounded by publication bias: positive findings (e.g., VPA’s synergistic effect with TMZ, CBD’s induction of tumor cell death) are more likely to be published, while negative or inconclusive results (e.g., lack of antitumor activity for GBP or PGB) are often underreported. This bias creates an overestimation of AEDs’ overall repurposing potential and obscures the true spectrum of their antitumor efficacy. Additionally, preclinical studies frequently use supra-therapeutic concentrations (e.g., CBD at 20–60 μM in vitro vs. clinical plasma concentrations of 1–10 μM) to demonstrate significant effects, but such findings are rarely contextualized with clinical feasibility, leading to an inflated perception of their translational value.

Additionally, the metabolic intervention, epigenetic regulation, and other effects of some AEDs lack tumor specificity, which may cause off-target effects on normal cells and induce potential risks. Furthermore, evidence mostly comes from preclinical studies and small-sample observational studies, and large-sample, multicenter, randomized controlled trials (RCTs) verifying their efficacy and safety are lacking, making it difficult to formulate unified clinical practice guidelines. These issues—heterogeneous evidence quality, uneven research focus, and publication bias—collectively undermine the reliability of current conclusions and highlight the need for standardized preclinical models, transparent reporting of negative results, and prioritization of AEDs with mechanistic plausibility and preliminary clinical hints for further investigation.

### 3.3. Future Perspectives

Future research on repurposing AEDs for CNS tumor therapy should build on current mechanistic insights while addressing existing limitations, focusing on four interconnected key directions:

1. Advance precision combination therapy guided by molecular typing and biomarkers: Match AEDs with tumor subtypes and validate predictive biomarkers to select responsive patients. Explore synergistic combinations of high-potential AEDs (VPA, LEV, CBD) with TMZ or other CNS tumor therapies, leveraging complementary mechanisms.
2. Develop tumor-specific targeted delivery systems. Utilize nanocarriers [179] or blood–brain barrier-penetrating technologies to enhance AED accumulation, addressing CBD’s subtherapeutic clinical concentrations and VPA’s off-target toxicity.
3. Deepen synergy with novel therapies. Integrate AEDs with immunotherapy to amplify TIME remodeling, combine with ferroptosis inducers to reinforce CBD’s oxidative stress effects, or pair with epigenetic drugs to enhance VPA’s HDAC inhibition.
4. Strengthen translational research. Conduct standardized preclinical studies using CNS tumor-specific models, design large-sample randomized controlled trials to validate survival benefits, and establish pharmacokinetic-pharmacodynamic correlations to optimize dosing while minimizing toxicities. These efforts will accelerate AEDs’ transformation from adjuvant agents for tumor-related epilepsy to core components of personalized CNS tumor treatment paradigms.

## 4. Conclusions

With diverse molecular mechanisms, AEDs have gradually evolved from agents that primarily control epilepsy to multifunctional agents with significant antitumor potential. By interfering with tumor progression through multiple pathways, they demonstrate significant promise for repurposing. Although challenges remain, these are expected to be addressed with advances in biology and delivery systems, potentially integrating AEDs into CNS tumor treatment paradigms. While challenges such as tumor heterogeneity, narrow therapeutic windows, and a lack of robust clinical evidence remain, these hurdles are anticipated be overcome through advances in molecular profiling, drug delivery technologies, and the execution of well-designed clinical trials. Ultimately it is expected that AEDs will be formally admitted to the treatment paradigm for CNS tumors, providing patients with safer and more effective treatment options.

## Figures and Tables

**Table 2:** Strength of antitumor evidence, mechanisms, and relevant CNS tumor types of discussed AEDs.

AED	Mechanisms	Relevant CNS Tumor Types	Strength of Evidence *
Valproic Acid (VPA)	Metabolic reprogramming, epigenetic regulation (HDAC inhibition), ERS-UPR regulation, TIME remodeling	Glioma Neuroblastoma	High
Levetiracetam (LEV)	Glutamate metabolism inhibition, carbonic anhydrase inhibition, ncRNA regulation, TIME remodeling	Glioma Glioblastoma	Medium
Lacosamide (LCM)	miRNA regulation	Glioma	Low
Carbamazepine (CBZ)	DNA methylation regulation	Glioma	Low
Phenytoin (PHT)	DNA methylation regulation	Glioma	Low
Diazepam (DZP)	Glycolysis inhibition	Glioma	Low
Acetazolamide (AZM)	Carbonic anhydrase inhibition, TIME regulation	Glioma	Low
Stiripentol (STP)	Glycolysis inhibition, TMZ resistance reversal	Glioblastoma	Low
Topiramate (TPM)	Carbonic anhydrase inhibition	Glioma	Low
Zonisamide (ZNS)	Carbonic anhydrase inhibition	Glioma	Low
Cannabidiol (CBD)	ERS-UPR activation, ion homeostasis disruption, TIME remodeling	Glioma (stem cells) Glioblastoma	Medium
Fenfluramine (FFA)	ERS-UPR balance modulation (Theoretical speculation)	Glioma (Theoretical speculation)	Low

* Evidence strength classification: “High” indicates studies with consistent and in-depth results covering in vitro, in vivo, and preliminary clinical levels; “Medium” indicates a certain number of studies with clear mechanisms but limited clinical validation or incomplete in vivo data; “Low” indicates few preliminary studies (mostly in vitro) or only theoretical mechanistic derivation, with insufficient experimental support.

## Data Availability

No new data were created or analyzed in this study.

## Abbreviations

The following abbreviations are used in this manuscript.

AED	Antiepileptic drug
CNS	Central nervous system
ER	Endoplasmic reticulum
ERS	ER stress
UPR	Unfolded protein response
TIME	Tumor immune microenvironment
VGSC	Voltage-gated sodium channel
CBZ	Carbamazepine
OXC	Oxcarbazepine
LCM	Lacosamide
VGCC	Voltage-gated calcium channel
ESX	Ethosuximide
GBP	Gabapentin
PGB	Pregabalin
GABA	γ-aminobutyric acid
PB	Phenobarbital
CZP	Clonazepam
STP	Stiripentol
VPA	Valproic acid
PER	Perampanel
AMPA	α-amino-3-hydroxy-5-methyl-4-isoxazolepropionic acid
LEV	Levetiracetam
BRV	Brivaracetam
SV2A	Synaptic vesicle glycoprotein 2A
TPM	Topiramate
FBM	Felbamate
FFA	Fenfluramine
5-HT	Serotonergic system
ESL	Eslicarbazepine
PHT	Phenytoin
LTG	Lamotrigine
PRM	Primidone
DZP	Diazepam
LZP	Lorazepam
TGB	Tiagabine
VGB	Vigabatrin
ZNS	Zonisamide
GABAAR	GABA-A receptor
NMDAR	N-methyl-D-aspartic acid receptor
AMPAR	α-amino-3-hydroxy-5-methyl-4-isoxazolepropionic acid receptor
GBM	Glioblastoma
GLUT	Glucose Transporter
MCT	Monocarboxylate transporter
PKM-2	Pyruvate kinase isozyme type M2
HK	Hexokinase
AZM	Acetazolamide
LDH	Lactate dehydrogenase
LDHA	Lactate dehydrogenase A
PFK	Phosphofructokinase
DHAP	Dihydroxyacetone phosphate
GA3P	Glyceraldehyde-3-phosphate
3PG	3-phosphoglycerate
2PG	2-phosphoglycerate
PEP	Phosphoenolpyruvate
NAD	Nicotinamide-Adenine Dinucleotide
TMZ	Temozolomide
SLC7A11	Solute carrier family 7 number 11
NMDA	N-methyl-D-aspartic acid
CA	Carbonic anhydrase
EMT	Epithelial–mesenchymal transition
CBD	Cannabidiol
CB1R	Cannabinoid receptor type 1
ROS	Reactive oxygen species
NRF2	Nuclear factor erythroid 2-related factor 2
IDH	Isocitrate dehydrogenase
FASN	Fatty acid synthase
MGMT	O (6)-methylguanine-DNA methyltransferase
mTOR	Mammalian target of rapamycin
DNAm	DNA methylation
SAM	S-adenosylmethionine
5mC	5-methylcytosine
DNMT	DNA methyltransferase
TET	Ten-eleven translocation family protein
LINE-1	Long interspersed element-1
Me	Methyl group
HDAC	Histone deacetylase
HDACi	HDAC inhibitor
CDKI	Cyclin-dependent kinase inhibitor
SOCS	Suppressor of cytokine signaling
ncRNA	Non-coding RNA
MALAT1	Metastasis-associated lung adenocarcinoma transcript 1
lncRNA	Long non-coding RNA
IRE1	Inositol requiring enzyme-1
PERK	Protein kinase-like endoplasmic reticulum kinase
ATF6	Activating Transcription Factor 6
BiP (GRP78)	78 kDa glucose-regulated protein
ATF4	Activating transcription factor 4
CHOP	C-EBP homologous protein
XBP1	X-box binding protein 1
TRPV	Transient receptor potential vanilloid 1
eIF2α	Eukaryotic Initiation Factor-2α
ERAD	ER-associated degradation
DDIT3	CHOP
TRIB3	Tibbles pseudokinase 3
SQSTM1	Squestosome 1
Hrd1	HMG-CoA reductase degradation protein 1
CB	Cannabinoid Receptor
Bcl-2	B-cell lymphoma-2
Bax	Bcl-2-associated X protein
PARP	Poly(ADP-ribose) polymerase
PINK1	PTEN-induced putative kinase protein 1
PRKN	Parkin
GPX4	Glutathione peroxidase 4
ALIX	ALG-2-interacting protein X
SEL1L	Suppressor/enhancer of Lin-12-like protein 1-like
VDAC1	Voltage-dependent anion channel 1
mPTP	Mitochondrial permeability transition pores
JNK1/2	Janus kinase 1/2
BECN1	Beclin-1
GAM	Glioma-associated macrophage
GSC	Glioma stem cell
TREM2	Triggering receptor expressed on myeloid cells 2
Tex	Terminal exhausted phenotype
NK	Natural killer
GM-CSF	Granulocyte-macrophage colony-stimulating factor
MDSC	Myeloid derived suppressor cell
JAK/STAT	Janus kinase/Signal transducers and activators of transcription
CAF	Cancer-associated fibroblast
IL-8	Interleukin-8
IDO	Indolamine-2,3-dioxygenase
VEGF	Vascular endothelial growth factor
FGF	Fibroblast growth factor
TGF-β	Transforming growth factor-β
p38/MAPK	p38 mitogen-activated protein kinase
TREM1	Triggering receptor expressed on myeloid cells 1
TLR	Toll-like receptor
Anti-PD-L1	Anti-programmed cell death ligand 1
IFN-γ	Interferon-γ
RCT	Randomized controlled trial
